# Resistance Mechanisms of *Saccharomyces cerevisiae* to Commercial Formulations of Glyphosate Involve DNA Damage Repair, the Cell Cycle, and the Cell Wall Structure

**DOI:** 10.1534/g3.120.401183

**Published:** 2020-04-16

**Authors:** Apoorva Ravishankar, Amaury Pupo, Jennifer E. G. Gallagher

**Affiliations:** Department of Biology, West Virginia University

**Keywords:** commercial formulations of glyphosate, herbicide resistance, *Saccharomyces cerevisiae*, cell wall, Sed1, Dip5, transcriptomics, whole-genome resequencing, in-lab evolutions

## Abstract

The use of glyphosate-based herbicides is widespread and despite their extensive use, their effects are yet to be deciphered completely. The additives in commercial formulations of glyphosate, though labeled inert when used individually, have adverse effects when used in combination with other additives along with the active ingredient. As a species, *Saccharomyces cerevisiae* has a wide range of resistance to glyphosate-based herbicides. To investigate the underlying genetic differences between sensitive and resistant strains, global changes in gene expression were measured, when yeast were exposed to a glyphosate-based herbicide (GBH). Expression of genes involved in numerous pathways crucial to the cell’s functioning, such as DNA replication, MAPK signaling, meiosis, and cell wall synthesis changed. Because so many diverse pathways were affected, these strains were then subjected to in-lab-evolutions (ILE) to select mutations that confer increased resistance. Common fragile sites were found to play a role in adaptation to resistance to long-term exposure of GBHs. Copy number increased in approximately 100 genes associated with cell wall proteins, mitochondria, and sterol transport. Taking ILE and transcriptomic data into account it is evident that GBHs affect multiple biological processes in the cell. One such component is the cell wall structure which acts as a protective barrier in alleviating the stress caused by exposure to inert additives in GBHs. Sed1, a GPI-cell wall protein, plays an important role in tolerance of a GBH. Hence, a detailed study of the changes occurring at the genome and transcriptome levels is essential to better understand the effects of an environmental stressor such as a GBH, on the cell as a whole.

Glyphosate-based formulations are among the most commonly used broad-spectrum herbicides around the world (Duke and Powles 2008) because of their low toxicity to mammals, high efficacy and affordability in comparison to other herbicides (Powles and Yu 2010; Duke 2018). Glyphosate acts by inhibiting the biosynthesis of aromatic amino acids, which is a product of the shikimate pathway. It targets the 5-enolpyruvylshikimate-3-phosphate synthase (EPSPS) enzyme, that converts phosphoenolpyruvate (PEP) to 3-phospho-shikimate to 5-enolpyruvylshikimate-3-phosphate (Amrhein *et al.* 1980; Powles and Preston 2006). Glyphosate resembles the transition state of the enzyme’s natural substrate and prevents the accumulation of 5-enolpyruvylshikimate-3-phosphate. Glyphosate binds EPSPS in plants inhibiting the shikimate pathway and the production of tryptophan (W), tyrosine (Y), phenylalanine (F), para-aminobenzoic acid, and coenzyme Q10 (Haderlie *et al.* 1977).

Organisms undergo different modes of adaptation to attain resistance to environmental stressors. In the case of glyphosate-based herbicides, the routes to attaining resistance are classified into two categories: 1. Target site associated resistance, and 2. Non-target site resistance (Yuan *et al.* 2007). Target site associated resistance is comprised of resistance mechanisms involving changes in the EPSPS gene and the shikimate pathway. Typically, either the glyphosate binding site in EPSPS is mutated or EPSPS is overexpressed. Non-target site resistance is achieved by changes occurring outside the shikimate pathway. One of the most commonly studied forms of non-target site resistance is vacuole related changes (Hereward *et al.* 2018; Moretti *et al.* 2017). Recent studies showed that proteins involved in glyphosate transport are one of the non-target resistance mechanisms resulting in glyphosate resistance (Rong-Mullins *et al.* 2017a). Some yeast strains encode an allele of the pleiotropic drug response protein, Pdr5, that results in resistance to a commercial formulation of glyphosate (Rong-Mullins *et al.* 2017a). The Pdr5 protein is involved in the transport of glyphosate out of the cell. Another non-target resistance mechanism comes from the protein Dip5, a glutamic acid and aspartic acid permease. Dip5 imports glyphosate, as glyphosate resembles glutamic acid and aspartic acid structurally. The deletion or downregulation of Dip5 by the addition of aspartic acid leads to glyphosate tolerance (Rong-Mullins *et al.* 2017a).

There are hundreds of glyphosate-based herbicides (GBHs) available in the market. The most challenging aspect of studying the effects of these GBHs is that the label lists the concentration of glyphosate present in the mixture but does not provide details regarding the additives or their concentrations. It is crucial to monitor and study these GBHs as a whole, along with their additives, as they alter the effectiveness of the principal component and other factors such as its extent of biodegradability (Mesnage *et al.* 2013). Some of the identified additives are polyoxyethylamines (POE), quaternary ammonium compounds and some heavy metals (Defarge *et al.* 2018; Nicolas Defarge *et al.* 2016; Nørskov *et al.* 2019). As the contributions of these additives to the toxicity of the herbicide have been shown to vary, they are now being studied in more detail (Jacques *et al.* 2019). The changes these additives inflict on the genome and transcriptome of organisms, would vary depending on the specific GBH.

Studying the synergistic effects of the active ingredient of these herbicides with their additives in different organisms, revealed effects such as apoptosis and necrosis in placental cells (Benachour and Séralini 2009), disruption of endocrine-signaling pathways (Gasnier *et al.* 2009; Romano *et al.* 2012), teratogenic effects in *Xenopus*, and chicken embryos (Paganelli *et al.* 2010), and even alteration of the digestive microbiome (Shehata *et al.* 2013; Krüger *et al.* 2014) to mention a few. The US EPA report stated that glyphosate is not carcinogenic to humans. While, the International Agency for Research on Cancer (IARC), released a controversial report declaring that it probably is carcinogenic to humans. This displays the need for further investigation of these chemicals. The one reason for the contradiction in these two reports can be narrowed down to the EPA relying on unpublished regulatory studies, that tested - glyphosate itself; however, the IARC selectively used peer-reviewed articles that studied glyphosate-based herbicides and carried out AMPA (aminomethylphosphonic acid) assays (Benbrook 2019).

The yeast cell wall provides structural support and is the first physical barrier of the cell that the herbicides encounter. The cell wall is 15–30% of the dry weight of the yeast cell (Orlean 1997). It is mainly comprised of an inner layer made of polysaccharides and an outer scaffold made of mannoproteins (Klis, *et al.* 2002; Orlean 2012; Stewart and Stewart 2018). The mannoproteins with β-1,3 glucans, and β-1,6 glucans, form the major components of the cell wall; with chitin forming non-covalent bonds with some glucans as a minor component. The cell wall is highly dynamic in nature and it can adapt to various physiological and morphological conditions (Aguilar-Uscanga and François 2003). The function of the cell wall is to stabilize internal osmotic pressure, protect the cell against mechanical injury and chemical stress, maintain the cell shape, and provide a scaffold for glycoproteins (Stewart and Stewart 2018). Some of these structural cell wall proteins are rich in serine and threonine, and they undergo a post-translational addition of a glycosylphosphatidylinositol (GPI) anchor which tethers them to the cell membrane when the GPI anchor inserts into the lipid bilayer (Nuoffer *et al.* 1991). Sed1 is one of many GPI-anchored mannoproteins in the cell wall-bound to a glucan. Cells in the stationary phase are found to have increased Sed1 levels (Shimoi *et al.* 1998) and also tend to be much more resistant to various environmental stressors (Werner-Washburne *et al.* 1993). The induction of these proteins occur on exposure to stress (Shimoi *et al.* 1998).

The goal of this research study was to establish if there is a difference between the effects of exposure to pure glyphosate and commercial formulations of glyphosate. If there was a difference, we wanted to further characterize the differences between a commercial formulation, based on the additives present and pure glyphosate. Results of initial studies led to the in-depth study of changes in the cell on exposure to one of the GBHs. *Saccharomyces cerevisiae* has a wide range of genetic variation within the species. Some of this variation pertains to the origin of these strains, in terms of their prior exposure to different stressors. In this study, yeast is used as a eukaryotic model to study the effect that glyphosate and the additives in commercial formulations, have on higher eukaryotes. Using the genetic variation in yeast we assessed the effects of GBHs on the transcriptome of different strains isolated from various environments such as vineyards, agricultural isolates, and clinical samples. Some of the strains used were sensitive to low concentrations of glyphosate exposure, whereas others were tolerant of much higher levels. This range of inherent sensitivities made it possible to carry out a comparative study to analyze the differences between the strains that confer their relative sensitivity or resistance.

Our results demonstrate that GBHs induce a pleiotropic response in cells exposed to it. They affected numerous pathways involved in various vital processes for the cell’s survival such as meiosis, DNA replication, cell wall proteins, MAPK and HOG signaling pathways, etc. The high osmotic response (HOG pathway) aids in the survival of the cell in case of cell wall stress (García *et al.* 2009). As the cell wall serves as the first physical barrier of the cell to encounter the GBHs, we focused on understanding the involvement of the cell wall, though many other critical pathways and mechanisms are altered on exposure to GBHs. Some cell wall proteins such as Sed1, when differentially expressed contributed to the tolerance of cells to GBHs. Another interesting aspect was the contribution of Ty elements to the adaptation of tolerance mechanisms against GBHs. Numerous genes that were flanked by Ty elements on either side, underwent gene duplication while developing resistance to GBHs. Many of these changes could have a cumulative response resulting in the cells’ developing resistance against GBHs.

## Materials and Methods

### Variations in growth phenotype

A semi-quantitative growth assay was carried out on exposure of AWRI1631, RM11, YJM789, and S288c, to different formulations of glyphosate-based herbicides. The two types of media used for the study was nutrient-rich (YPD) and nutrient minimal (YM) solid media. The rich media is comprised of yeast extract, peptone, and dextrose; as for the minimal media, it consists of yeast-nitrogen base and 2% dextrose. The minimal media was supplemented with aromatic amino acids, namely 20 μg/ml tryptophan (W), 30 μg/ml tyrosine (Y) and 50 μg/ml phenylalanine (F). It was also supplemented with 100 μg/ml aspartic acid (D) for certain conditions. The growth assay was performed as previously described in (Rong-Mullins *et al.* 2017a), as follows. The optical density of the saturated cultures was measured and used to standardize the number of cells for each strain. 10-fold dilutions were carried out in a 96-well plate and using a pinner, transferred onto agar plates containing media. The cells were exposed to the glyphosate-based herbicides and the concentration of glyphosate was standardized to 1.0% in rich and 0.15% in minimal media respectively. The different formulations used were, Compare and Save (CAS), WeedPro (WP), RoundUp Super Concentrated (RU-SC), Credit 41 (Cr41) and pure glyphosate as a control.

Quantitative growth analysis was carried out using a TECAN M200, automatic plate reader (Rong-Mullins *et al.* 2017b). All cultures were started at an optical density of 0.1 at 600nm to maintain uniformity among strains. The growth of cells in liquid media was measured every 1 hr at 600nm under shaking conditions (200 rpm). Along with Credit41 and pure glyphosate, the cells were treated with 5mM calcofluor white, to measure the progression of their growth over 50 hr. Doubling time was calculated from each replicate growth curve, and the differences between conditions assessed by pairwise *t*-test. For the calculation of the doubling time: for each culture the time range of log growth was selected. The curves in the selected ranges were adjusted to the function log (OD) = k*t + C, where OD:optical density, t: time, k: slope, C: intercept. From the slope the doubling time is calculated as T= ln2/k.

### Gene expression analysis

RNA was extracted from S288c and RM11 cells grown in minimal media, with and without Cr41 treatment. The same was carried out in minimal media supplemented with WYF. The cells were treated with 0.25% Credit41 for 90 min with 5 replicates of each condition. The samples were then washed, and the total RNA was extracted using hot phenol method (Ausubel *et al.* 1995). Paired-end cDNA libraries were built using the RNA extracted using Epicenre’s ScriptSeq Yeast kit. The sequencing was performed using 76bp paired-end reads on the Illumina HiSeq platform resulting in 5- 8.5 million read pairs.

Salmon v0.13.1 was used to estimate the transcript level abundance from the RNA seq-read data (Patro *et al.* 2017). To do so, indexing of the S288c reference transcriptome was first carried out. This index was used by Salmon against each sample to generate quant.sf files containing the length, abundance in terms of Transcripts Per Million (TPM) and the estimated number of reads for each transcript. The differential expression of genes between the two strains and each condition was calculated using DESeq2 v1.8.1. The cells grown in minimal media, treated with Cr41, was analyzed against cells grown in minimal media without Cr41 treatment. The cells grown in minimal media supplemented with WYF, were similarly normalized. DESeq2 was also used to generate a sample to sample distance map (Fig S4). The program clusterProfiler v3.10.0 was used to carry out KEGG Pathway Enrichment Analysis, to identify the pathways involved, based on the clustering of genes.

### Data availability

Strains and plasmids are available upon request. The authors affirm that all data necessary for confirming the conclusions of the article are present within the article, figures, and tables. Gene expression data are available at GENO with the accession number: GSE135473. Supplemental material available at figshare: https://doi.org/10.25387/g3.12132924.

### Flow cytometry

To measure the cell cycle arrest on Credit41 and pure glyphosate exposure, S288c and RM11 cells were subjected to flow cytometry after exposure. The cultures were started and allowed to grow to mid-log phase from an overnight culture. Once the cells reached mid-log phase, they were treated with 0.25% Credit41 and pure glyphosate, in triplicate with and without supplementing WYF in minimal media. Cells were collected at multiple time points (0 min, 30 min, 90 min, 3 hr, and 6 hr) over the next 6 hr. The cells were maintained in log-phase throughout the collections, by replenishing with fresh media as needed. The cells were harvested and fixed in 70% ethanol for 2 days (Haase and Reed 2002). The cells were then washed and treated with RNAse solution for 8-12 hr at 37°. The RNAse solution comprised of a mixture of 2mg/ml RNAse A, 50mM Tris pH 8.0, and 15mM sodium chloride that had been boiled for 15 min and cooled to room temperature. The cells were then treated with protease solution (5 mg/ml pepsin and 4.5 μl/ml concentrated HCl) for 20 min at 37°. The cells were then stored in 50 mM Tris pH 7.5 at 4°, until the day of the analysis. Right before flow cytometry analysis, the cells were sonicated at low levels to separate cells. 50ul of the sonicated mixture was transferred into 1ml of 1uM Sytox Green in 50ml Tris pH 7.5. The cells were analyzed on an LSRFortessa, using the FITC channel. The results were analyzed, and the percentage of cells in each stage of the cell cycle was estimated using FCS Express 6.0. The analysis was carried out using the multi-cycle DNA histogram and ‘+S order = 1 model’ was selected based on the lowest Chi-square value.

### Whole-genome sequencing of In-Lab evolutions (ILEs)

In-lab evolutions were carried out by exposing cells to Credit41 over a period of time (six passages ∼15days) until a resistant population was isolated. Two sensitive (S288c and YJM789), and two resistant (RM11 and AWRI1631) strains were used for this study. The cells were evolved in minimal media with 0.25% Cr41, with and without supplementing WYF. The cells evolved in rich media were treated with 1.0% Cr41. A control group was evolved in media in the absence of Credit41 to account for mutations that occur due to the procedure, rather than the Credit41 treatment. A single colony was used to start a saturated overnight culture, that was then used as a starter culture to inoculate the different conditions in triplicate. Each passage was grown 2-3 days in media till saturation. After which 1% of the culture was transferred into fresh media. The cells were subjected to 6 passages and tested for resistant populations by plating 10-fold dilutions on solid media. The resistant populations were streaked on plates to isolate single colonies. The resistant colonies were selected based on size and shape. They were then passaged for 2 passages in media lacking Credit41, to ensure the resistance did not rely solely on epigenetic mechanisms. The genomic DNA was then extracted using the 96 well genomic DNA extraction kit, and the phenol-chloroform extraction technique was also used (Hirt 1967).

The whole-genome sequencing data for all 4 strains were aligned to S288c reference sequence (release R64-2-1) by creating an index and using gatk-4.1.1.0 to carry out the alignment. Samtools was used to convert the SAM files to BAM files, from which duplicates were removed. Using gatk HaplotypeCaller, variant calling was carried out to identify all the SNPs and generate vcf files. Using the generated vcf files, PCA variants were identified using pcadapt as described in (Gauch *et al.* 2019) (https://data.mendeley.com/datasets/ts5syfw38r/draft?a=e2170360-459e-4a8c-b1b3-d89f4b530fdf) (doi 10.17632/ts5syfw38r).

## Results

### Variation in growth in the presence of glyphosate-based commercial formulations

Four genetically diverse haploid strains with well-annotated genomes (S1 Table (Song *et al.* 2015)) were chosen to test for differences in growth when exposed to different glyphosate-based herbicides. RM11 is a vineyard strain (Mortimer *et al.* 1994; Török *et al.* 1996) and AWRI1631 is descended from commonly used strain for commercial wine manufacturing (Borneman *et al.* 2008). YJM789 is derived from a clinical isolate, isolated from an immunocompromised AIDS patient (McCusker *et al.* 1994; Tawfik *et al.* 1989). The last strain included was a commonly used laboratory strain with an S288c background (GSY147) (Engel *et al.* 2014; R K Mortimer and Johnston 1986; Wenger *et al.* 2010). The provenance of the agricultural strains was difficult to determine retroactively. Both the agricultural strains used were isolated in the 1980s. Commercial glyphosate has been available since the 1970s and it is likely that these strains were exposed to a GBH. We exposed these strains to commercial formulations of glyphosate-based herbicides on solid media, with pure glyphosate as a control. We exploited this variation in strain backgrounds, to decipher the effects of the different commercially available formulations of glyphosate-based herbicides. There are a large number of formulations available, many of which contain a mixture of multiple active ingredients such as pelargonic acid, diquat, imazapic, and other chemicals. The GBH selected for this study were chosen because they were labeled as containing glyphosate as the sole active ingredient in the herbicide, hence the variation observed is the clear effect of the putatively inert additives.

**Figure 1:**
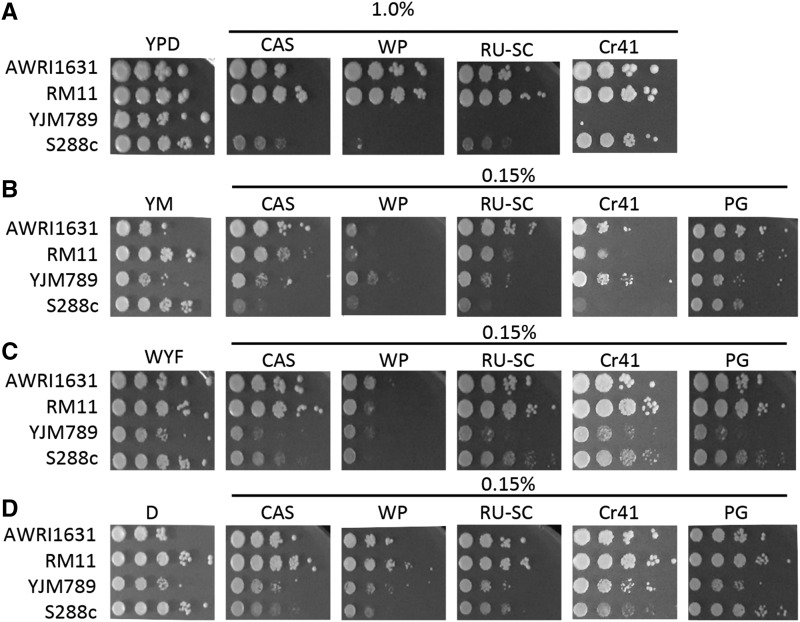
Contribution of genetic variation of different yeast strains to glyphosate resistance. Serial dilutions (1:10) of haploid AWRI1631, RM11, YJM789, and S288c were grown on the following media A. YPD with 1.0% of different commercial formulations of glyphosate-based herbicides (GBHs). B. Minimal media (YM) with 0.15% of different GBHs and PG. C. YM supplemented with aromatic amino acids (WYF) with 0.15% of different GBHs and PG. D. YM supplemented with aspartic acid (D) with 0.15% of different GBHs and PG. Compare and save (CAS); WeedPro (WP); RoundUp Super concentrated (RU-SC); Credit 41 (Cr41) and pure glyphosate (PG).

To test the effects on different yeast strains, the concentrations of GBHs used in rich and minimal media were established by performing a growth assay using a dose curve. The concentration of glyphosate in each GBH was optimized for each type of media to highlight the variation in growth across the strains (Rong-Mullins *et al.* 2017a). Rich media (YPD) contains all the amino acids, while minimal media (YM) only provides carbon and nitrogen sources. Four commercially available GBHs were chosen, *i.e.*, Compare and Save (CAS), Weed Pro (WP), RoundUp Super Concentrate (RU-SC), and Credit 41 (Cr41). They were all diluted to contain the same amount of glyphosate for growth assays. When grown with GBHs on YPD, AWRI1631 and RM11 continued to grow in the presence of 1% glyphosate in all the formulations (Figure 1A). Whereas YJM789 and S288c displayed a growth defect, and S288c was sensitive to all the commercial formulations of glyphosate but extremely sensitive to the exposure of WP. YJM789 was incapable of growth on exposure to any of the commercial formulations. Pure glyphosate (PG) is not soluble at 1% in solid media, hence growth was not tested on YPD plates. On minimal media (YM), where cells synthesize their own amino acids from the nitrogen base provided, ammonium sulfate, in this case, there was greater variation between yeast responses to the formulations, especially in the case of WP where all the strains were sensitive, but more or less the pattern was consistent among the other formulations, with S288c showing maximum sensitivity and AWRI1631 showing resistance to a larger extent (Figure 1B). Glyphosate targets the aromatic amino acid pathway and supplementing the minimal media with tryptophan, tyrosine, and phenylalanine (WYF) is predicted to bypass the inhibition of the shikimate pathway and restore growth (Rong-Mullins *et al.* 2017a). However, rescue with WYF in the presence of different GBHs was variable. Growth improved relative to the growth observed on unamended YM; although, the cells did not fully recover in any of the commercial formulations (Figure 1C). AWRI1631 and RM11 growth inhibition was less compared to YJM789 and S288c. S288c was the most affected by all the formulations. All the strains were sensitive to WP and supplementing WYF did not show considerable rescue from the effects of this formulation. This observation indicates that even when bypassing the effects of the aromatic amino acid biosynthetic pathway, there are factors in the GBHs influencing the cell growth, that do not pertain to the active ingredient which is common among them and these vary from one formulation to another. Dip5 is an amino acid permease that is linked to glyphosate transport into the cell (Rong-Mullins *et al.* 2017a). It is downregulated in the presence of excess aspartic acid (Hatakeyama *et al.* 2010), and adding back aspartic acid did rescue the cells to an extent but not in the case of S288c (Figure 1D). The addition of excess aspartic acid to cells treated with pure glyphosate rescued the growth inhibition in all the strains. YJM789 showed a significant rescue of growth on treatment with Cr41 when supplemented with aspartic acid. The addition of aspartic acid could possibly be decreasing the amount of glyphosate entering the cell through Dip5. The growth inhibition resulting from exposure to WP was alleviated to a greater extent on the addition of aspartic acid in comparison to WYF, but not completely. A similar extent of the growth inhibition was induced by Cr41, CAS and RU-SC. To consider a formulation that is representative of a phenotype similar to most of the commercial formulations, Cr41 was used for all further experiments.

### GBH-response mechanisms have a strain and condition-dependent pattern

**Figure 2:**
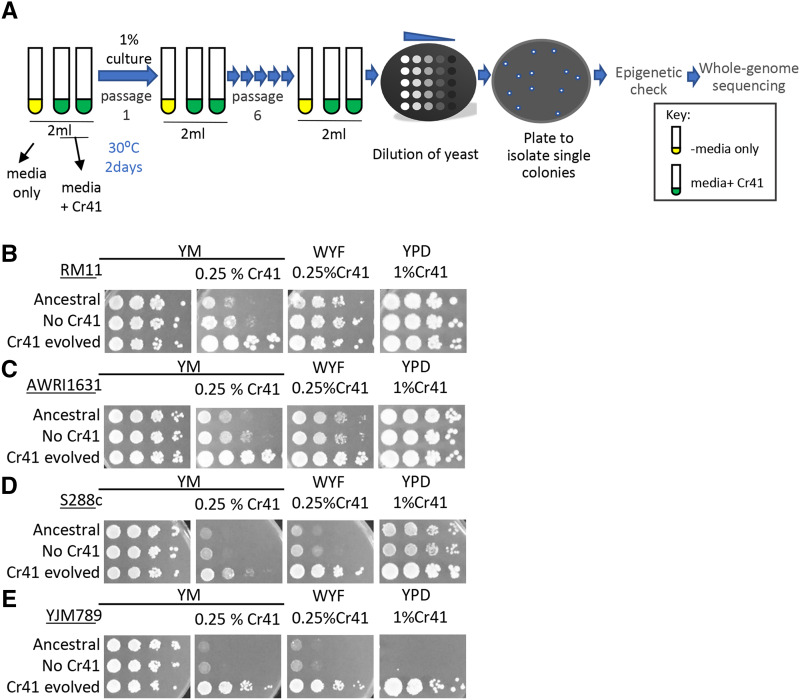
Selection of strains resistant to commercial glyphosate formula, Credit41 by In-lab evolutions (ILEs). A. Outline of In-lab evolutions (ILEs) methodology: The cells were grown in media with (green) and without (yellow) Credit41 for 2 days at 30° on the shaker. 1% of the culture was transferred to fresh media for 6 passages. Serial dilutions of the cultures of the sixth passage were plated on solid media. The resistant lines were plated on solid media to isolate single colonies. The genomics DNA was extracted and submitted for Illumina sequencing. B. Serial dilutions of haploid RM11, strain evolved without and with Cr41, on media with Cr41. C. AWRI1631, D. S288c, E. YJM789.

Genomic sequence data indicate tens of thousands of SNPs between these four strains (Wei *et al.* 2007; Doniger *et al.* 2008; Borneman *et al.* 2008). To explore how genomes, change and permit adaptation to high levels of Cr41, the yeast were serially passaged in media containing Cr41 by performing In-Lab-Evolutions (ILEs). Three biological replicates were passaged in three different types of media: 1) YM, 2) YM supplemented with WYF and 3) YPD media. These were supplemented with 0.25% Cr41 (*i.e.*, 0.25% glyphosate content) in minimal media, and 1% in rich media respectively (Figure 2A). The strains were diluted through six passages by transferring 1% culture to fresh media. Once the resistant populations were identified, single colonies were isolated. These strains were then released from the selective pressure for two passages and then the Cr41 resistance was confirmed (Figure 2B-E). This step was performed in order to minimize the possibility that the resistance was entirely due to epigenetic mechanisms, as epigenetic mechanisms cannot be detected through whole-genome sequencing. The resistant cells sequenced for each strain along with the condition from which each strain was evolved, are listed in S2 Table. The ILEs selected a large number of SNPs as well as duplicated regions. The synonymous mutations were filtered out and only the genes containing non-synonymous mutations were taken into consideration. A total of 148 genes (S3 Table) accumulated at least one non-synonymous SNP within the coding region among all the sequenced strains treated with Cr41. The genes that accumulated more than one SNP and/or indel in any of the samples from different strains were prioritized and focused on for this study. Dip5, in part, transports glyphosate into the cell as shown in our previous study (Rong-Mullins *et al.* 2017a), and contained SNPs in three of the sequenced samples (Fig S1).

**Figure 3:**
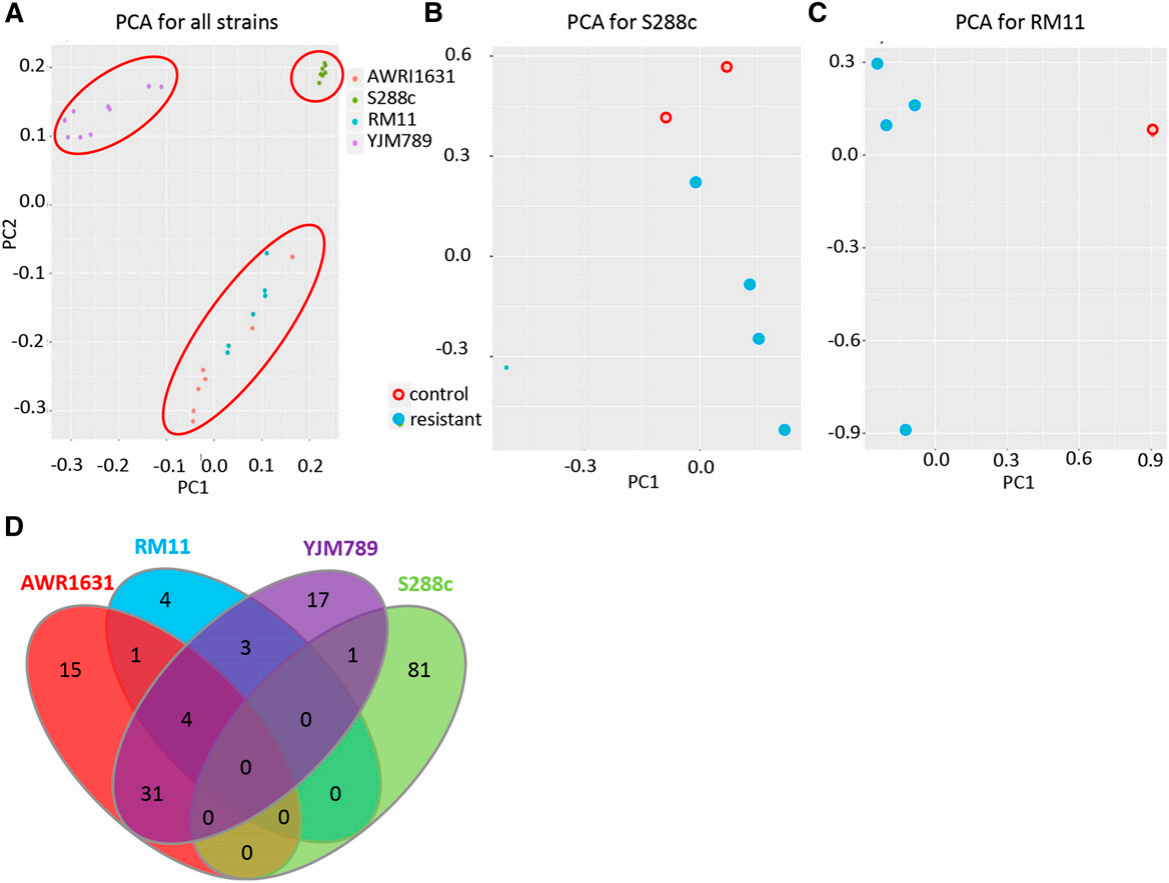
Whole-genome analysis of In-lab evolutions (ILEs). A. PCA of SNPs of various samples from all 4 strains, AWRI1631, S288c, RM11 and YJM789 B. PCA of SNPs identified among the S288c ILE samples C. PCA of SNPs identified among the RM11 ILE samples D. Venn-diagram representing all the affected genes *i.e.*, those that contained CNV as well as those that accumulated SNPs, in each strain.

Principal Component Analyses (PCA plots) were plotted for each sample taking all the SNPs into consideration in order to identify the major sources of variation. This was done to ensure that the main sources of variation corresponding to the biological conditions and was not a mere effect of experimental bias. An R-package, pcadapt (Luu *et al.* 2017) visualized the patterns that naturally exist between the strains. Hence, resistant strains (AWRI1631 and RM11) clustered together and so did the sensitive strains (S288c and YJM789), when the data from all the conditions were pooled together (Figure 3A). The underlying background genetic differences of the strains dominated the genome comparisons. Further analysis of the strain-specific PCA plots showed that the clustering of the controls *vs.* the Cr41 treated samples in S288c was based on PC2 (Figure 3B), and PC1 did the same in the case of RM11 (Figure 3C). The separations among the YJM789 and AWRI1631 samples were not as evident (Fig S2).

Sections of the genome containing as few as two, to as many as a hundred genes increased in copy number (CN) as shown by copy number variation (CNV) analysis. All the strains in this study were haploid, and only nonessential genes could have resulted in a CN of zero. In this analysis, any genes that were partially duplicated were excluded under the assumption that the change in copy number is associated with synteny or the other genes present in their immediate surroundings (S4 Table). Any gene that underwent a CNV in a treated cell but also had an increase in CN in any control sample, passaged without Cr41, was filtered out. It was apparent that there is an underlying connection between the strain and the condition (*i.e.*, YM, YM with WYF or YPD) it was evolved under. In the case of YJM789, CNVs occurred only in cells that were evolved in minimal media with WYF. S288c had the most genes (81 genes) that underwent CNV while RM11 did not have any, which was one of the most resistant strains. Some of the S288c genes that underwent CNV were found in all the conditions, but many of which were found in cells evolved in minimal media supplemented with WYF. Though these genes underwent duplication in a specific condition (*i.e.*, WYF), they did not belong to a single pathway. Their functionality ranged from mitochondrial maintenance (*i.e.*, *e.g.* *MRX14*, *MRLP1*) to biosynthesis of secondary metabolites such as inositol (*INO2*). This led to the hypothesis that the route used to attain resistance was dependent on the media type. Hence, if a certain strain was evolved in YM, it may not be resistant to the GBH in YPD. To confirm this hypothesis, semi-quantitative growth assays of the evolved strains were carried out in different combinations of media conditions. The strains evolved in rich media did not confer cross-resistance in minimal media. However, those evolved in minimal media and WYF, confer cross-resistance (Fig S3).

Many regions that underwent CNV were flanked by fragile sites, mainly consisting of transposable elements (Lemoine *et al.* 2005) and one long regulatory ncRNA, *ICR1* (Table 1). Fragile sites are defined as regions of the genome that make it difficult for the cell to undergo replication and sometimes result in chromosome breakage. Studies in other organisms such as *Caenorhabditis elegans* and human cells have shown evidence of DNA damage in these fragile sites (Koller *et al.* 2012; Jacques *et al.* 2019). Past studies lack conclusive evidence to declare that Ty elements are involved in fragile site rearrangements, resulting in CNV of certain regions. However, there is evidence to prove that these elements are involved in processes such as translocations, deletions, etc. (Dunham *et al.* 2002; Roeder and Fink 1980). Most of the regions undergoing an increase in copy number and being flanked by Ty elements were found in S288c cells, evolved in the presence of Cr41. All the genes that contained non-synonymous SNPs or underwent CNV (S4 Table) were referred to as “affected genes” because they were affected by the Cr41 treatment (Figure 3D). The regions that underwent CNV were large sections containing multiple genes from different pathways such as MAP kinase/ Hog1 pathway, mitochondrial genes, DNA damage repair pathways, spindle formation, metal transporters, cell wall, and cell membrane.

**Table 1:** Regions with CNVs flanked by fragile sites in the ILEs

Chromosome	Name	Type	start	end	width	Sample	Copy Number
chrII	YBLCdelta7	Ty1 LTR	197016	197320	305	AWRI-YM-1	CN2
chrII	YBLWsigma1	Ty3 LTR	197714	198054	341	YJM789-WYF-1	CN2
chrIV	YDLCdelta1	Ty1 LTR	434423	434739	317	AWRI-YM-1	CN2
chrIV	YDRCdelta7	Ty1 LTR	645502	645835	334	GSY147-WYF-2	CN2
chrIV	YDRCdelta8	Ty1 LTR	651086	651419	334	GSY147-WYF-3	CN2
chrIV	YDRCsigma1	Ty3 LTR	651420	651503	84	GSY147-YPD	CN2
chrIV	YDRCdelta9	Ty1 LTR	651682	651980	299	GSY147-YM-2	CN3
chrIV	YDRWsigma2	Ty3 LTR	668096	668436	341	GSY147-WYF-2	CN2
chrIV	YDRWdelta10	Ty1 LTR	668543	668784	242	GSY147-WYF-3	CN2
chrV	YERCdelta23	Ty1 LTR	492695	492833	139	GSY147-YM-2	CN2
chrVIII	YHRCdelta16	Ty1 LTR	549306	549637	332	AWRI-YPD	CN2
chrIX	*ICR1*	lncRNA	393884	397082	3199	GSY147-WYF-3	CN2

### Transcriptome analysis revealed differential expression of many genes in the sensitive *vs.* resistant strains

**Figure 4:**
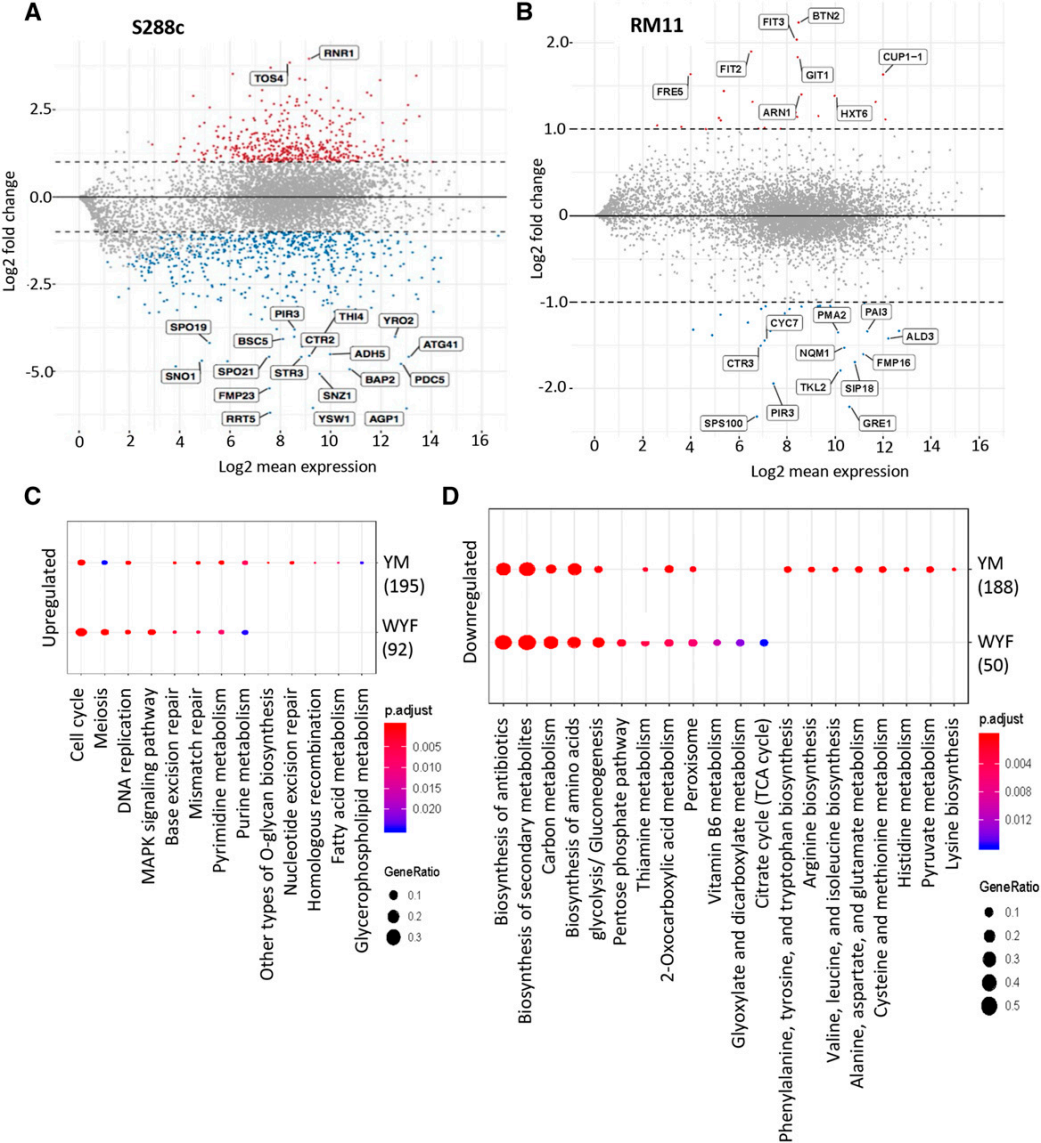
Gene expression analysis of S288c and RM11 on exposure to glyphosate (Credit41) in minimal media A. Dot-plot of significantly up and down-regulated genes in S288c B. in RM11 C. KEGG Pathway Enrichment analysis of upregulated genes in S288c, identifying the different pathways involved D. KEGG Pathway Enrichment analysis of downregulated genes in S288c, identifying the different pathways involved.

To determine if there was a correlation between the genes that underwent changes in the ILEs and those that are differentially expressed on exposure to Cr41, an RNAseq experiment was carried out. The RNA was extracted and sequenced from cells grown in two types of media, minimal media and media supplemented with WYF, in the presence and absence of Cr41 therefore, making it a total of four conditions. The strains were treated with Cr41 in two different conditions, minimal media (YM) and media supplemented with WYF. The genes that were differentially expressed in YM treated with Cr41, were normalized to RNAseq of cells grown in YM without Cr41 treatment. This same form of comparison was carried out between cells grown in media supplemented with WYF, in the presence and absence of Cr41 treatment. This study was carried out in two media conditions, as this would help identify the genes differentially expressed based on the effects caused by the commercial formulation, as well as those with respect to the additives alone, in case of the WYF supplemented media. The cells treated in minimal media with Cr41 were analyzed to characterize the response to glyphosate and the additives in the commercial formulation; the cells treated with Cr41 in minimal media supplemented with WYF were analyzed to isolate the effects of the additives while bypassing effects that occur due to inhibition of the aromatic amino acid pathway. The transcriptome analysis was performed on two strains, one of which was sensitive (S288c) to Cr41 exposure, and the other resistant (RM11). To decide on which two strains to consider for this study, we also factored in that the two strains had the most variation in the ILE study in terms of the number of SNPs and the genes that underwent CNV. S288c, the sensitive strain treated with Cr41, had a much higher number of differentially expressed genes as compared to RM11 in the RNAseq (Figure 4A). In YM, 1100 genes (S5 Table), and 438 genes (S6 Table) in media supplemented with WYF, were differentially expressed in S288c when exposed to Cr41 with a final concentration of 0.25% glyphosate. In RM11 treated with Cr41, only 58 (S7 Table) and 53 (S8 Table) genes were differentially expressed in minimal media and media supplemented with WYF, respectively. RM11 differentially expressed fewer genes in both conditions *i.e.*, minimal media and media supplemented with WYF (Figure 4B), the genes expressed seemed to have a higher dependence on the condition in which they were treated. The Cr41 treated cells were analyzed in both media conditions in each strain to identify the commonalities in the response mechanisms in the presence and absence of WYF. S288c had 280 common genes that were differentially expressed when Cr41 was applied in YM and WYF media while in RM11 only 28 genes were differentially expressed (Table S9). Of these genes, the ones that were common between the two strains did not highlight any particular pathway or defense mechanism. KEGG pathway enrichment analysis of the differentially expressed genes in S288c revealed that many of these genes corresponded to pathways involved in the cell cycle, meiosis, DNA replication, and MAPK signaling pathways, especially in WYF (Figure 4C and 4D). Among many others, *SED1* is one of the cell wall genes that was differentially regulated along with a few transposable elements in S288c, but no SNPs were found in the ILEs. The downregulated genes mainly associated with biosynthesis of secondary metabolites and amino acids in both minimal media and media supplemented with WYF.

### S288c cells arrested in G1 on exposure to Cr41 but not pure glyphosate

**Figure 5:**
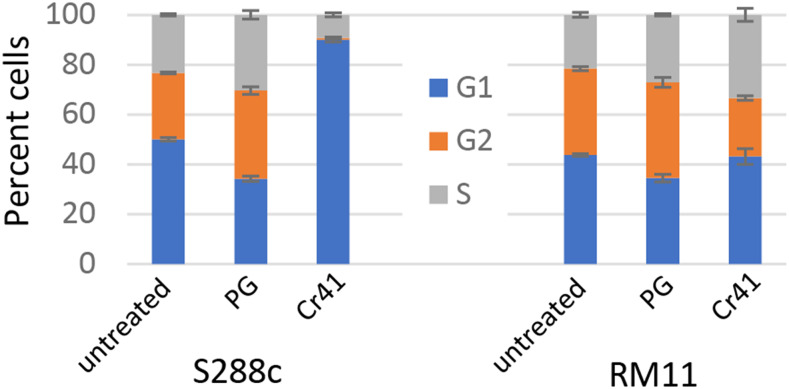
Cell cycle distribution on treatment with glyphosate and a commercial formulation of glyphosate. Distribution of the cell cycle stages of S288c and RM11 cells on exposure to Credit41 (Cr41) and pure glyphosate (PG) for six hours.

The RNAseq data showed upregulation of the expression of cell cycle regulator genes encoding proteins that are G1 phase regulators such as Rad53, Cdc28, Nrm1, and Swi4 (Bertoli *et al.* 2013). Increased expression of these genes suggested that cells were arrested in G1 on exposure to Cr41. To ascertain if it was the glyphosate itself or a cumulative effect of all the additives in GBHs that caused cell cycle arrest, cells treated with Cr41 and pure glyphosate were subjected to flow cytometry. RM11 and S288c were grown to log-phase and then the asynchronous populations were exposed to 0.25% pure glyphosate and the equivalent of the amount of glyphosate from Cr41 (Fig S5). Within 30 min of Cr41 exposure, 70% of the population arrested in G1 and remained the same over the course of 6 hr while the culture density was maintained consistently (Figure 5). This was not the case in the untreated and pure glyphosate treated cells. In these two cases, the populations were distributed across the G1, G2, and S phases. RM11 cells did not show any changes in the cell cycle with respect to the two treatments. Only S288c, the strain that was sensitive to Cr41 exposure, underwent G1 arrest almost immediately on exposure to Cr41. One of the common reasons for G1 arrest in *S cerevisiae* is DNA damage signaling and repair (Bartkova *et al.* 1997; Fitz Gerald *et al.* 2002). The increased expression of *RAD53* also highlights the probability of DNA damage dependent G1 arrest.

### sed1∆ mutants are highly sensitive to Cr41 exposure

**Figure 6:**
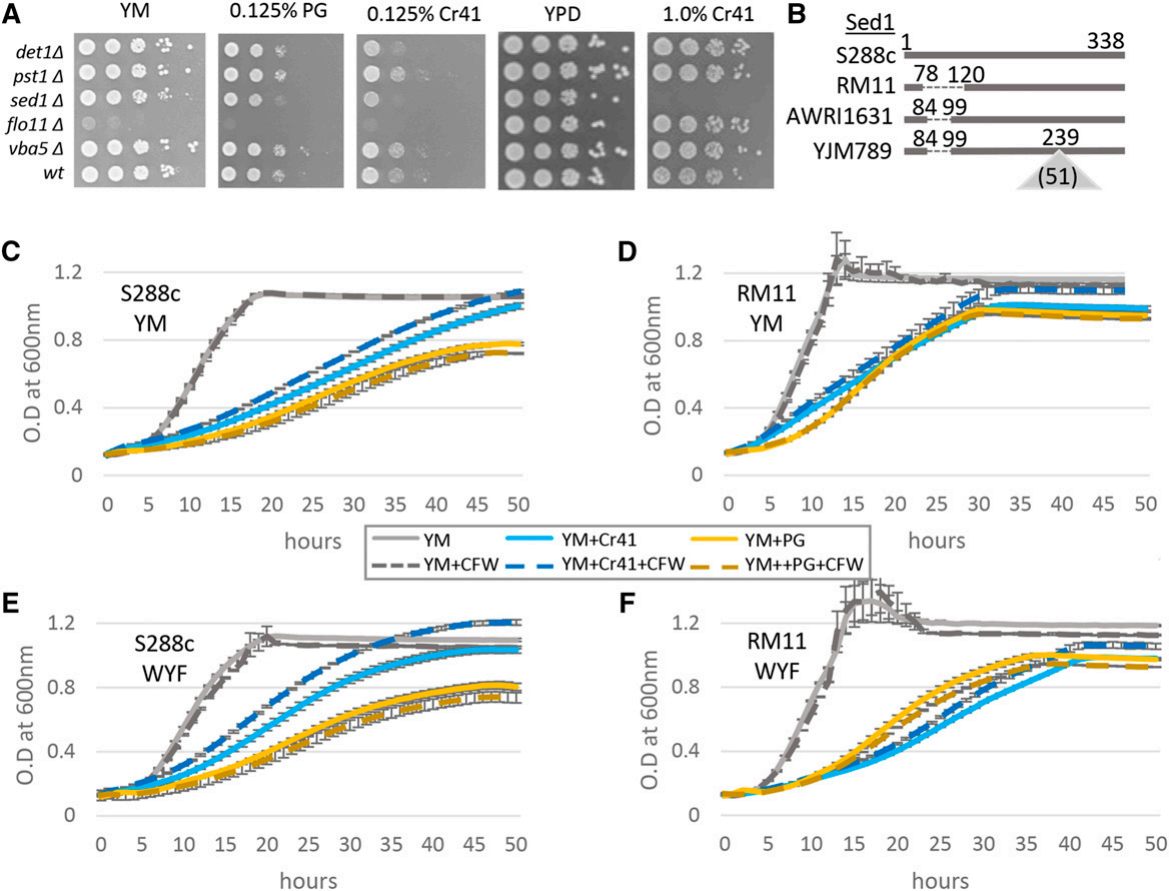
Growth assay on exposure to calcofluor white and glyphosate-based herbicides. A. Semi-quantitative growth assay of cell wall mutants. Cells were grown for three days. B. Protein alignment of Sed1. Deletions in reference to the S288c are a dashed line and insertions are denoted by a triangle. Amino acid numbers are in reference to S288c with the number of inserted amino acids in parenthesis. Quantitative growth assays of cells grown in minimal media when exposure to calcofluor white (CFW, dashed line), Credit41 (Cr41, blue) and pure glyphosate (PG, yellow). OD_600_ was measure for 50 hr in an automatic plate reader. C. S288c in YM, D. RM11 in YM. Yeast were grown in minimal media supplemented with tryptophan (W), tyrosine (Y), phenylalanine (F). E. S288c in WYF, F. RM11 in WYF.

The genes commonly affected in the ILE study and differentially expressed in the RNAseq analysis are involved in various pathways, including functions ranging from DNA damage repair, cell wall proteins, mitochondrial proteins, MAPK related proteins to meiosis (S4 Table). The BY4741 (also an S288c derived strain) knockout collection was used to test the growth phenotype of the different genes that were identified in the ILEs or the RNAseq, as representatives from some of these pathways (Figure 6A). Among the different cellular processes and components affected, the cell wall was the one chosen to carry out further investigation. Five genes were selected for further characterization, all of which encoded proteins in the cell wall or cell membrane. Det1 plays an integral role in intracellular sterol transport (Sullivan *et al.* 2009). *PST1* encodes a GPI protein that works with Ecm33 to maintain cell wall integrity (Pardo *et al.* 2004). The presence of Emc33 could be the cause of the lack of sensitivity in *pst1**∆* mutants on exposure to Cr41. *SED1* is a gene that is expressed as a major stress-induced cell wall glycoprotein (Shimoi *et al.* 1998). *FLO11* encodes a GPI-anchored cell wall protein, whose transcription is regulated by the MAPK pathway (Rupp *et al.* 1999). The *flo11**∆* mutants showed growth defects in minimal media. *VBA5* is a paralog of *VBA3*, and it codes for a plasma membrane protein that plays a role in amino acid uptake (Shimazu *et al.* 2012). Not all the knockouts changed growth in response to Cr41 exposure as many of the genes may work in unison to have an overall effect on resistance to the treatment. Another possibility is the specific mutation could have a gain of function effect, which cannot be mimicked by using a knockout collection. One of the cell wall genes that had a definitive response was *SED1*. *SED1* was found in both the ILE data with its copy number doubled, and expression decreased by 1.475 log2 fold in the transcriptome analysis in GBH sensitive S288c. The *sed1**Δ* mutant was extremely sensitive to Cr41 exposure in both rich and minimal media (Figure 6A). The *sed1**∆* mutant was found to be more sensitive to Cr41 exposure as compared to PG. This supports our hypothesis, components other than glyphosate in the GBHs contribute to the sensitivity of the cells by affecting cell wall proteins. In the S288c ILE strain that developed resistance to Cr41, *SED1* underwent duplication in all media conditions, *i.e.*, minimal media with and without WYF, and rich media. Downregulation of *SED1* gene in YM in S288c (sensitive strain) could contribute to the sensitivity of the cells to Cr41. No mutations were found in *SED1* from the ILE sequencing but there are several insertions and deletions found in the parental strains of RM11, YJM789, and AWRI1631 in reference to S288c (Figure 6B). Sed1 is a stress-induced structural GPI (glycosylphosphatidylinositol) cell wall glycoprotein (Shimoi *et al.*1998), hence its sensitivity to Cr41 due to the additives could be affecting the integrity of the cell.

### Calcofluor white (CFW) alleviates growth inhibition of S288c caused by Cr41 exposure

The analysis of affected genes in the evolved strains and the differentially expressed genes gave rise to certain genes that are associated with the cell wall. To test if the cell wall played an important role in the effectiveness of Cr41, one sensitive and one resistant strain that was treated with Cr41 was exposed to Calcofluor white (CFW) (Figure 6C-F). CFW is known for its property of inducing cell wall stress, as it is a chitin antagonist and results in increased deposition of chitin, making the cell walls thicker (Liesche *et al.* 2015; Roncero and Duran 1985). A quantitative liquid growth assay was carried out using S288c and RM11. The growth of RM11 was inhibited by pure glyphosate and Cr41 treatment to an extent. Treating the RM11 samples with CFW did not result in drastic effects on the alleviation of growth inhibition in YM, for Cr41 and PG. The significance of growth inhibition alleviation caused by CFW treatment was calculated for each strain by conducting pairwise t-Test analysis (Table S10), calculating the slope and doubling time (Table S11) using the Holm method to adjust the p-value (Fig S6). Different letters above the boxes mean statistically significant differences in the means (p adjusted > 0.05 in a pairwise *t*-test, with p adjusted by Holm). The statistical grouping of the growth curves only compares one characteristic of the growth curves. Yeast were grown to saturation in YM and then diluted into fresh media in the microtiter plate. As cells exit stationary phase, the length of that lag phase can vary. There is usually variation within the population which can only be determined through single cell assays not conducted here. The growth rate is measured as the maximum slope during log phase (exponential). During normal growth cells begin the diauxic shift as nutrients begin to be become depleted and cells reenter stationary phase. The final concentration of the culture is yet another characteristic of a growth curve. Therefore, cells that reenter the cell cycle later but have a steeper slope in their log phase will have a line that overlaps with cells that started growing sooner but do not grow as fast. For example, RM11 grown in YM+PG *vs.* YM+Cr41 (Figure 6D and S6B). The individual traces for each biological replicate for the growth curves that illustrate the differences between conditions (Fig S6). Unlike the other strains, S288c grew slower with PG compared to Cr41.

To decipher if there was a genetic link to this response, other strains that are genetically close to S288c were also tested with PG and Cr41. All strains closely related to S288c showed a higher sensitivity to PG compared to Cr41 over the first 50 hr (Fig S7). Sed1 is polymorphic in S288c compared to YJM789, AWRI1631 and RM11 (Figure 6B). It is possible that variation in multiple genes contribute to variation in import and export of PG and Cr41 in S288c which would account for the increased sensitivity to PG. Treating S288c cells in YM exposed to pure glyphosate with CFW showed an increase in growth inhibition (Figure 6C-E). This could be due to CFW treatment effecting the cell wall structure of the cells. However, CFW alleviated the growth inhibition caused by Cr41 in S288c cells. Cells treated with CFW are known to have increased cell wall volume by about 30% and the wall/cell ratio also increases significantly (Liesche *et al.* 2015). The increase in cell wall thickness could be the main contributing factor to the mild rescue of cells observed in Cr41 treated cells. This observation indicated that the additives in Cr41 along with glyphosate have a cumulative effect on the cell wall of sensitive strains such as S288c, which was alleviated by CFW treatment. The increase in cell wall thickness on CFW treatment could help the cells withstand the effects of Cr41, also it may reduce the amount of Cr41 entering the cell. However, treating cells exposed to only pure glyphosate with CFW does not result in alleviation of growth inhibition. The data imply that CFW is not rescuing cells from the glyphosate in Cr41 but is alleviating the effects of the additives on the cell wall.

## Discussion

Glyphosate-based herbicides are most commonly used around the world because of the specificity of glyphosate, acting solely on the aromatic amino acid pathway, that is absent in humans and many other eukaryotes (Reeds 2000; Tanney and Hutchison 2010). The additives and surfactants present in the commercial formulations are chosen due to their intrinsic inert and non-toxic properties (Myers *et al.* 2016). There are variations from one GBH to the other in terms of these additives, which result in different degrees of the effectiveness of the herbicide (Defarge *et al.* 2018). The supposed non-active ingredients that are added in GBHs enhance the potency of glyphosate and were not inert as seen by changes in our transcriptome analyses. The study of all the changes occurring within the cell to make the herbicide more effective, due to the presence of additives and surfactants, is crucial. The additives in the GBHs along with other functions play a key role in the entry of the active ingredient into the cell (Arand *et al.* 2018). The cell wall being the outermost barrier of the cell, the effectiveness of GBHs vary based on the structure and composition of the cell wall along with the alleles of genes encoding different mannoproteins that the cell wall contains.

When the concentrations and combinations of additives change, this also changes their cumulative effect on the cell’s growth as shown by the semi-quantitative growth assay carried out with different GBHs. Yeast from different environments varied in their growth when exposed to different GBHs (Barney *et al.* 2020). The strains chosen for this study have been isolated from different environments, with one of them likely to have prior exposure to a GBH (Mortimer *et al.* 1994). Based on the nutrients available to the cells and those that are absent, genes belonging to different pathways are up/down regulated and this leads to changes in the proteins expressed. This could be one possible explanation for a strain such as YJM789 that is extremely sensitive to GBHs in YPD, but the sensitivity is alleviated in minimal media. The agricultural isolates (RM11, AWRI1631) were more resistant to GBHs and glyphosate, compared to the laboratory strain and the clinical isolate (YJM789) which were more sensitive. The agricultural isolates may have come in contact with glyphosate or similar herbicides in the past, which could have led to the development of resistance mechanisms based on past exposure. The lab strain and clinical isolate were not in environments commonly exposed to GBHs, making them less likely to have been exposed in the past, contributing to their sensitivity.

The genetic variation between these strains was used, to gain a better understanding of the effects of the GBHs as a whole at the genome and transcriptome levels. In case of some stressors, adaptation can occur through a specific route depending on the biological processes that undergo changes, when encountering the stressor (Parsons *et al.* 2006; Zakrzewska *et al.* 2011). Response to GBHs is a polygenic trait, as the multiple genes determine the phenotype observed with exposure to this stressor. Hence, as seen in the ILE strains, adaptation can result from small modifications that occur in many different genes as a cumulative effect, as well as one particular gene, could have had a significant contribution as shown in the case of *sed1**∆* mutants. Effects of each individual mutation found in the ILEs were not tested in further detail. Because of the number of mutations selected in the ILEs strains, the contribution to GBH resistance of each one would need to be determined empirically. Large sections of the genome were found to have undergone CNV, many of which were flanked by Ty elements and ncRNAs. The genetic variation between the strains is not only pertaining to the gene sequences but also the response and resistance mechanisms observed on exposure to GBHs. RM11 being a Cr41 resistant strain, had 10-fold less genes that were differentially expressed. This pattern was also observed in the ILEs where no genes underwent increase in copy number in RM11. This could be due to the limited distress the cells were in due to their ability to tolerate GBH exposure. On the other hand, S288c had many genes that were differentially expressed as they could be trying different mechanisms to deal with the detrimental effects GBHs have on the cell.

Regulatory elements have been shown to play an important role in combatting stressors that the cell encounters (McClintock 1984). *S. cerevisiae* has five families of LTR retrotransposons, and they comprise about 3% of the entire genome (Garfinkel 2005). Mutations in certain DNA repair genes can result in the expression of common fragile sites, which when induced often undergo translocations, deletions, duplications, etc. (Casper *et al.* 2002). The ILE strains contained mutations in genes involved in many pathways, some of which were contributors to DNA repair mechanisms (such as *RAD53*, *UME6*, and *MSH3*) as well. Any of these alterations could have induced fragile site expression, in turn resulting in duplication of the genes present between two Ty elements. Having the Ty elements in these regions provided the opportunity for these genes to undergo duplication, and those with a beneficial effect could be retained as the population progressed. Over the course of serial passaging of these strains, the duplicated regions along with the effects of SNPs in some of the genes may have provided the advantage that the cells needed to overcome sensitivity to Cr41. An increase in the copy number of a gene is a mechanism that could be used by a cell to gain resistance to an environmental stressor (Hill *et al.* 2013; Ford *et al.* 2015). Regions of the genome containing multiple genes undergoing a copy number increase is a commonly observed phenomenon (Ford *et al.* 2015). The duplication of one or more of these genes could be providing a fitness benefit that increases the cell’s ability to combat the stressor. Regions such as common fragile sites aid in the rearrangement or modification of these sites. Identifying the genes that underwent gene duplication while adapting to exposure of a GBH to understand the processes being affected by exposure to these herbicides. In this study, we validated the effects of one such gene *SED1*. Future studies will focus on other biological processes that are affected, for example, mitochondrial function. It was also interesting to see the role CNV itself played in combatting the effects of a stressor by undergoing gene duplications and the role of Ty elements, which facilitated in this process. These results provide support in deciphering the role of Ty elements, an important genome adaptation mechanism on encountering an environmental stressor.

Glyphosate-based herbicides affect regions of the cell apart from the aromatic amino acid pathway. The cell wall was one of the many pathways affected. The cell wall is a mode of entry into the cell and a vital structure in maintaining the osmotic integrity of the cell during stress. Changing the combinations or concentrations of additives in an herbicide alters the mode of entry of the active component into the cell. In our previous study, we have shown that proteins such as Dip5, the aspartic and glutamic acid permease (Rong-Mullins *et al.* 2017a) are involved in the import of some glyphosate into the cell; Dip5 was identified by comparing S288c and YJM789 genomes through a QTL analysis. Polymorphisms in the protein-coding regions changed the sensitivity of the yeast strain (Rong-Mullins *et al.* 2017a). In the QTL analysis, only genes that were genetically different between these strains were detected. Therefore, there could be other permeases/transporters that also regulate glyphosate, that were not identified in the QTL analysis as they were genetically identical in these two strains. The effect of pleiotropic drug response genes was more evident in rich media. *PDR5* is the most polymorphic gene in yeast (Guan *et al.* 2010). Pdr5 shares 96% amino acid similarity between S288c and YJM789 (Guan *et al.* 2010). Whereas Pdr5 is 99.7% similar between S288c and RM11 (Rong-Mullins *et al.* 2017a), which may have led to the masking of its contribution to this study. There may have been other proteins that were not identified in the previous study due to the high similarity of those proteins in YJM789 and S288c.

To assess which cell wall proteins contributed significantly to Cr41 resistance, the knockout collection was used to test all the cell wall genes that were either affected in the ILEs or were differentially regulated in the RNA seq. Among these, a gene could have an overarching contribution to attaining resistance which would be identified on testing its knockout, or it could incrementally contribute to a cumulative effect of multiple genes that underwent changes, in which case its role may not be as clear in knockout studies. This knockout-screening approach led to the observation that Sed1 is an important contributor to the cell’s resistance to GBHs. Sed1 is a GPI-cell wall protein that is highly expressed when the cells are in stationary phase and is a required protein for cells in this phase when they are under stress (Shimoi *et al.* 1998). Sed1 is not very highly conserved across strains, and a 120bp deletion in RM11 led to a similarity of only 87.87% between the S288c and RM11 alleles. Like Pdr5, the role of Sed1 appears to be primarily aimed at dealing with the effects of the additives in Cr41. Like the *pdr5* mutant, the *sed1* knockout was very sensitive to Cr41 when present in higher levels in YPD. There is other evidence that points to a role of the cell wall in responding to Cr41. QTL analysis of RM11 and S288c strains linked *TIR1*, a gene encoding a cell wall mannoprotein, Cr41 resistance (Ravishankar *et al.* 2020). The MAPK pathway along with Hog1 coregulate genes involved in maintaining the integrity of the cell membrane and cell wall. Several duplicated genes (*FUS3*, *STE5*, *BMH2*, and *AFR1*) in the evolved strains belong to pathways that are regulated by Hog1 MAP kinase pathway. This pathway is activated under conditions of hyperosmotic stress (Schüller *et al.* 1994) and is usually accompanied by differential expression of various GPI-cell wall proteins (Kapteyn *et al.* 2001). The GBH as a whole could be inducing hyperosmotic stress resulting in the activation of the Hog1 MAP kinase pathway which could also contribute to arresting in G1 phase (Escoté, *et al.* 2004).

In this study, we highlight the importance of studying different GBHs to gain a better understanding of all the pathways affected when exposed to Cr41 and to recognize the various non-target adaptation mechanisms. Humans have proteins that have structural and functional similarity to those found in yeast. As this herbicide is used so extensively on produce that is used for human consumption (Beckie *et al.* 2020), it is important that we understand the effects of the complex chemical mixtures we are being exposed to. Recent studies have shown the presence of glyphosate and its metabolites in urine samples in humans (Mills *et al.* 2017; Parvez *et al.* 2018), though the levels of glyphosate humans are exposed to through food consumption are much lower than those used in this study to treat yeast cells. Without analyzing the effects at the molecular level, it is difficult to predict if there will be any long-term effects of human ingestion of GBHs. Lack of acute effects is not necessarily an indication that there would not be any effects after long-term exposure. Hence, it is crucial to study the effects of different GBHs to be able to regulate the use of additives and surfactants.
